# Correction: Bayesian hierarchical modeling of joint spatiotemporal risk patterns for Human Immunodeficiency Virus (HIV) and Tuberculosis (TB) in Kenya

**DOI:** 10.1371/journal.pone.0352668

**Published:** 2026-06-25

**Authors:** Verrah A. Otiende, Thomas N. Achia, Henry G. Mwambi

The Data Availability statement for this article is incorrect. The correct statement is: All data and analysis code supporting this study are publicly available at https://github.com/Verrah/HIV-TB-Kenya-Spatiotemporal (permanently archived at https://doi.org/10.5281/zenodo.19680403).
