# Supplementary material for: Bayesian hierarchical modeling of joint spatiotemporal risk patterns for Human Immunodeficiency Virus (HIV) and Tuberculosis (TB) in Kenya
Source: PLoS One. 2020 Jul 2;15(7):e0234456. doi: 10.1371/journal.pone.0234456 (PMC7332062; doi:10.1371/journal.pone.0234456)
Supplement: S2 Text — (DOCX) [file pone.0234456.s002.docx]

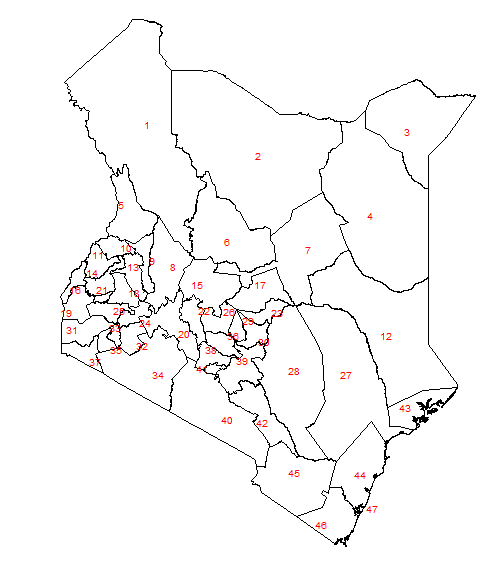


| [1] | Turkana | [26] | Nyeri |
| --- | --- | --- | --- |
| [2] | Marsabit | [27] | Tana River |
| [3] | Mandera | [28] | Kitui |
| [4] | Wajir | [29] | Kirinyaga |
| [5] | West Pokot | [30] | Embu |
| [6] | Samburu | [31] | Homa Bay |
| [7] | Isiolo | [32] | Bomet |
| [8] | Baringo | [33] | Nyamira |
| [9] | Keiyo-Marakwet | [34] | Narok |
| [10] | Trans Nzoia | [35] | Kisii |
| [11] | Bungoma | [36] | Murang'a |
| [12] | Garissa | [37] | Migori |
| [13] | Uasin Gishu | [38] | Kiambu |
| [14] | Kakamega | [39] | Machakos |
| [15] | Laikipia | [40] | Kajiado |
| [16] | Busia | [41] | Nairobi |
| [17] | Meru | [42] | Makueni |
| [18] | Nandi | [43] | Lamu |
| [19] | Siaya | [44] | Kilifi |
| [20] | Nakuru | [45] | Taita Taveta |
| [21] | Vihiga | [46] | Kwale |
| [22] | Nyandarua | [47] | Mombasa |
| [23] | Tharaka |  |  |
| [24] | Kericho |  |  |
| [25] | Kisumu |  |  |
