# Supplementary material for: Bayesian hierarchical modeling of joint spatiotemporal risk patterns for Human Immunodeficiency Virus (HIV) and Tuberculosis (TB) in Kenya
Source: PLoS One. 2020 Jul 2;15(7):e0234456. doi: 10.1371/journal.pone.0234456 (PMC7332062; doi:10.1371/journal.pone.0234456)
Supplement: S3 Text — (DOCX) [file pone.0234456.s003.docx]

| **Code** | **County** | **2012** | **2013** | **2014** | **2015** | **2016** | **2017** |
| --- | --- | --- | --- | --- | --- | --- | --- |
| 1 | Turkana | 951148 | 985389 | 1020863 | 1057614 | 1095688 | 1135133 |
| 2 | Marsabit | 308988 | 315167 | 321471 | 327900 | 334458 | 341147 |
| 3 | Mandera | 1321085 | 1437340 | 1563826 | 1701443 | 1851170 | 2014073 |
| 4 | Wajir | 852523 | 927545 | 1009169 | 1097976 | 1194597 | 1299722 |
| 5 | West Pokot | 570078 | 590601 | 611862 | 633889 | 656709 | 680351 |
| 6 | Samburu | 249014 | 257979 | 267266 | 276888 | 286856 | 297183 |
| 7 | Isiolo | 152065 | 155106 | 158208 | 161372 | 164600 | 167892 |
| 8 | Baringo | 617748 | 639986 | 663026 | 686895 | 711623 | 737242 |
| 9 | Keiyo-Marakwet | 411414 | 426224 | 441569 | 457465 | 473934 | 490995 |
| 10 | Trans Nzoia | 910404 | 943179 | 977133 | 1012310 | 1048753 | 1086508 |
| 11 | Bungoma | 1756338 | 1800246 | 1845252 | 1891383 | 1938668 | 1987135 |
| 12 | Garissa | 802447 | 873063 | 949892 | 1033483 | 1124429 | 1223379 |
| 13 | Uasin Gishu | 994269 | 1030062 | 1067145 | 1105562 | 1145362 | 1186595 |
| 14 | Kakamega | 1788339 | 1833048 | 1878874 | 1925846 | 1973992 | 2023342 |
| 15 | Laikipia | 443914 | 459895 | 476451 | 493604 | 511373 | 529783 |
| 16 | Busia | 525603 | 538743 | 552212 | 566017 | 580168 | 594672 |
| 17 | Meru | 1439317 | 1468104 | 1497466 | 1527415 | 1557964 | 1589123 |
| 18 | Nandi | 837248 | 867389 | 898615 | 930965 | 964480 | 999201 |
| 19 | Siaya | 896491 | 915318 | 934539 | 954165 | 974202 | 994660 |
| 20 | Nakuru | 1782793 | 1846973 | 1913464 | 1982349 | 2053713 | 2127647 |
| 21 | Vihiga | 597267 | 612199 | 627504 | 643191 | 659271 | 675753 |
| 22 | Nyandarua | 625349 | 635355 | 645521 | 655849 | 666342 | 677004 |
| 23 | Tharaka | 387691 | 395445 | 403354 | 411421 | 419649 | 428042 |
| 24 | Kericho | 843223 | 873579 | 905028 | 937609 | 971363 | 1006332 |
| 25 | Kisumu | 1031241 | 1052897 | 1075008 | 1097583 | 1120632 | 1144166 |
| 26 | Nyeri | 727384 | 739022 | 750847 | 762860 | 775066 | 787467 |
| 27 | Tana River | 261573 | 269159 | 276964 | 284996 | 293261 | 301766 |
| 28 | Kitui | 1074695 | 1096189 | 1118113 | 1140475 | 1163284 | 1186550 |
| 29 | Kirinyaga | 553808 | 562669 | 571672 | 580819 | 590112 | 599554 |
| 30 | Embu | 547808 | 558764 | 569940 | 581339 | 592965 | 604825 |
| 31 | Homa Bay | 1025797 | 1047339 | 1069333 | 1091789 | 1114716 | 1138126 |
| 32 | Bomet | 805248 | 834236 | 864269 | 895383 | 927616 | 961011 |
| 33 | Nyamira | 636739 | 650110 | 663763 | 677702 | 691933 | 706464 |
| 34 | Narok | 946167 | 980229 | 1015518 | 1052076 | 1089951 | 1129189 |
| 35 | Kisii | 1226411 | 1252166 | 1278461 | 1305309 | 1332720 | 1360707 |
| 36 | Murang'a | 988553 | 1004369 | 1020439 | 1036766 | 1053355 | 1070208 |
| 37 | Migori | 976174 | 996673 | 1017603 | 1038973 | 1060792 | 1083068 |
| 38 | Kiambu | 1702453 | 1729692 | 1757367 | 1785485 | 1814053 | 1843078 |
| 39 | Machakos | 1165826 | 1189143 | 1212926 | 1237184 | 1261928 | 1287166 |
| 40 | Kajiado | 764246 | 791759 | 820262 | 849792 | 880384 | 912078 |
| 41 | Nairobi | 3509911 | 3643287 | 3781732 | 3925438 | 4074605 | 4229440 |
| 42 | Makueni | 938667 | 957440 | 976589 | 996121 | 1016043 | 1036364 |
| 43 | Lamu | 110632 | 113840 | 117141 | 120538 | 124034 | 127631 |
| 44 | Kilifi | 1209109 | 1244173 | 1280254 | 1317381 | 1355585 | 1394897 |
| 45 | Taita Taveta | 310147 | 319142 | 328397 | 337920 | 347720 | 357804 |
| 46 | Kwale | 708131 | 728666 | 749798 | 771542 | 793917 | 816940 |
| 47 | Mombasa | 1023488 | 1053169 | 1083711 | 1115139 | 1147478 | 1180755 |

Source: Kenya National Bureau of Statistics (P.O. Box 30266-00100, Nairobi-Kenya; telephone: +254-20-317583; www.knbs.or.ke)
