# Supplementary material for: Bayesian hierarchical modeling of joint spatiotemporal risk patterns for Human Immunodeficiency Virus (HIV) and Tuberculosis (TB) in Kenya
Source: PLoS One. 2020 Jul 2;15(7):e0234456. doi: 10.1371/journal.pone.0234456 (PMC7332062; doi:10.1371/journal.pone.0234456)
Supplement: Attachment — Submitted filename: Response to Reviewers.docx [file pone.0234456.s007.docx]

# Reviewer 1

# Overview:

Overall, the authors followed some of the reviewer comments in the data description and methodology sections to improve this version of the manuscript. However, the conclusions and interpretations are still unclear and the figures presented remain misleading. In the results section, many of the plots claim to be showing relative risk, but in the text of the document it is not described how those relative risk values are calculated, and the figure labels do not clarify. Plots of the overall estimated relative risk in each region over time should be shown. Plots of relative risk need to have a different color scheme than currently shown since the current scheme is not informative. More description of what shared, disease-specific, and combined spatial and temporal trends/patterns are in relation to the model parameters needs to be added in the text and not just in the figure labels. As currently presented, the results section is difficult to follow and it remains unclear if the conclusions are supported by the data.

# Revision

The script has been revised accordingly to address the reviewer’s comments.

# Comment 1:

The first sentence should be deleted. It begins with “the spatiotemporal modeling of multiple diseases simultaneously” and ends by reordering those same words as “space-time analysis to model multiple diseases simultaneously.” The Abstract can begin with “The simultaneous spatiotemporal modeling of multiple related diseases strengthens inferences by borrowing information between related diseases.”

# Revision:

[Lines 18 - 19]: The first sentence adopts the recommendation from the reviewer.

# Comment 2:

The sentence on lines 50-51 can be deleted. “The latest and most recent extension is the joint spatiotemporal modeling of multiple diseases.” The sentence does not add to the discussion in that paragraph and “latest and most recent” is not necessarily true as the authors cite in the subsequent paragraph papers that do joint spatiotemporal modeling going back to 2004, 2006, and 2008.

# Revision:

[Line 51]: The statement "latest and most recent extension …" has been replaced with "another extension … "

# Comment 3:

Line 81. Change “These model maps” to “Maps generated from these models”.

# Revision:

[Lines 81 - 82]: Adopted the reviewer's suggestion

# Comment 4:

Line 103. “provides” should just be “provide”.

# Revision:

[Line 103]: Adopted the reviewer's suggestion

# Comment 5:

The added discussion about the relationship between HIV and TB helps address why one might jointly model these diseases, however it would benefit from a description of the raw data. A data summary or a map of the raw data for example.

# Revision:

[Lines 100 - 108] We have included some discussion on the TB - HIV situation in Kenya as observed by previous researchers as a motivation for further studying these two diseases.
[Lines 164 - 168] We have also included another section under the methods section after the data source sub-section to describe the variables in the study: HIV observed, TB observed, and the population estimates per county per year

# Comment 6:

Lines 99-100. The causality inferred by this statement is not clear. Surely one disease does not cause the other, but it can make one more susceptible to the other. This should be stated more clearly.

# Revision:

[Lines 99 - 101]: The statement has been stated more clearly

# Comment 7:

Line 121-122. Change “The population density has remained on the increase…” to “The population density has continued to increase…”

# Revision:

[Line 122 - 123]: Adopted the reviewer's suggestion

# Comment 8:

Line 164-165. Change to “We formulated a statistical model that applied spatiotemporal methods to the modeling of HIV and TB simultaneously.” This phrasing more accurately describes what the authors are doing which is using existing methodology in a new setting.

# Revision:

[Line 165]: Adopted the reviewer's suggestion

# Comment 9:

Line 167 and 168. Change the word “mapped” to “modeled” in both instances. Model results are what are being shown in a map.

# Revision:

[Lines 168 - 169]: Adopted the reviewer's suggestion

# Comment 10:

Line 181. Although the elaboration improves the paper, some aspects of the description and notation used for the standardization process remain unclear. Is N the same for each county? I would assume not, but that point is not entirely clear in the way that it is described. The current notation N=P/T implies that P and N are the same for each county. Indices using s and t could help clarify this point to know if N changes per county and/or over time.

# Revision:

[Line 182]: The notation and descriptions used for the standardization process are clarified.

# Comment 11:

Line 184. Technically P_{dst} would not need d in this instance, but it could in general be there if the two diseases had different susceptible populations.

# Revision:

[Line 185]: Adopted the reviewer's suggestion

# Comment 12:

It seems like the shared spatial and the disease specific spatial terms being in the same model may not all be identifiable individually. The figures seem to suggest this as well. In the Richardson et al paper that the methods are based upon, there is only a beta_{2s} parameter and the beta_{1s} is the reference category as specified on pages 392 and 393 of their paper. This should be addressed.

# Revision:

Richardson et al use an asymmetric formulation for their model as their interest was to determine the differential risk patterns, that’s the reason they used the beta_{i} as the female differential from the shared component. In our paper, we are using the shared component approach to separate the underlying risk of each disease into a shared component (common to both diseases) and disease-specific component for each disease. The estimated spatial patterns presented for the shared and disease-specific components are distinct and show no sign of identifiability problem. We imposed a sum-to-zero constraints on both the spatial and temporal random effects to minimize any identifiability problem on the intercept. Sum-to-zero constraints are considered as guaranteeing model identifiability.

# Comment 13:

Line 223. Please write out how the weighted matrix Q is used in the random walk of order 1.

# Revision:

# [Lines 240 - 251]: Adopted the reviewer's suggestion

# Comment 14:

Lines 225-226. Please specify whether the normal distribution is being parameterized in terms of the variance or precision. The fact that alpha has a value of 0 makes it seem like this is a precision parameter, while the scaling parameters are referenced as sigma^2 which makes it seem like a variance. Also, what value was used for sigma^2 in the prior?

# Revision:

[Lines 252 - 253] * We have specified the normal distribution is parameterized in terms of the variance, we have also expressed the precision parameter and explained the parameters correctly. We have also defined improper flat priors for the alpha which means it is not a probability distribution on its own but yields a well-defined posterior.

# Comment 15:

Line 226. Why are the scaling parameters not restricted to be positive? If I am understanding correctly, they cannot equal 0 since you use 1/delta, but the prior is centered at 0 which is confusing.

# Revision:

[Lines 254 – 259] We have restricted the scaling parameters to positive by including the expression delta > 0 and kappa > 0. Kappa and delta are symmetric around zero on their log-scale

# Comment 16:

Lines 245-246. It is reported that there is statistical significance at the 5% level as depicted by the credible intervals. Is this for both the intercept (alpha) and the precision (tau) parameters? Please include the delta and kappa parameters as well. The precision terms should not be include 0 anyway since that is a boundary. It would be informative to talk about the size of the precision terms. For instance, it is interesting that the shared component parameters for beta have larger precision than lambda does. This would suggest that there is very little variance in the shared parameter relative to the disease specific parameter.

# Revision:

[Lines 275 - 283] We have adjusted our explanation accordingly. For the intercepts (alpha) - it is evident that the values are significantly different from zero (as depicted by the credible intervals) implying that the overally (nationwide) disease-specific relative risk is above 1, which implies the country is general is still at a high risk for both infections. For the precision (tau) parameters, we have presented the variance measures instead of the precision values. The percentage of the variation expresses what each of the parameters contributes to the explained variability of HIV and TB. From this we are able to see that the shared components explain the most variability (comparative variation in the shared parameter relative to the disease specific parameters). Both the shared spatial and components dominate the model and cannot be left out of the model.

# Comment 17:

Figure 5. This plot could be improved by adding error bars for the 95% credible intervals. The authors are interpreting that the combined temporal trend plot of HIV risk is lower in 2012 and 2013, but 2015 and after it is TB that has higher risk. Error bars that do or do not overlap would better demonstrate the risk difference.

# Revision:

Figure 5: we have adopted the suggestion from the reviewer

# Comment 18:

Figure 6. The red category is for only a probability equal to 1. There are no regions showing this red color. It would be more informative to have the ranges as 0-0.25, 0.26-0.50, 0.51-0.75, 0.76-1.00.

# Revision:

Figure 6: We have adopted the suggestion from the reviewer. We also wish to emphasize that the figure is designed for a comparative illustration. The main information that authors were seeking to showcase was a transition of joint infection across Kenya. The authors were keen to present the spatiotemporal trajectory of the joint risk

# Comment 19:

Figure 7. The upper bound of the legend is 1.00 in the left panel. Is this correct since panel a is supposedly showing a relative risk? In the figure on the right, there is a typo in the lowest probability values. What is the justification for breaking probability into three unequal sized bins?

# Revision:

We have removed figure seven because it is confusing

# Comment 20:

Lines 269-270. Figure 6 is interpreted as “over the years the joint incidence risk reduced progressively with all spatial regions having a relative risk less than 1.” Figure 6 is showing probabilities, not relative risk, so this interpretation is wrong.

# Revision:

[Line 306 - 308] We have adjusted to interpret the probabilities and not the relative risk

# Comment 21:

Lines 272-273. Why are there disease specific parameters in the joint disease risk?

# Revision:

We have removed figure seven because it is confusing

# Comment 22:

I suggest changing the color schemes between the RR maps and the probability maps to avoid confusion.

# Revision:

Adopted the reviewer’s suggestion.

# Comment 23:

It is unclear what is being plotted in Figure 7 because it claims to be joint disease risk, but the label includes disease specific parameters. To address the significance of the shared parameters, you should look at posterior probabilities of lambda and xi (the shared parameters) being different from 0.

# Revision:

We have removed figure seven because it is confusing

# Comment 24:

How are relative risks determined to be statistically significant? If congruence is purely visual it should be interpreted as descriptive. If congruence is based on posterior means there should be some predetermined threshold and posterior means that differ by less than the threshold would be defined as congruent.

# Revision:

Our description for spatial congruence is areas of HIV high risks were found to be also high-risk areas for TB (converse is not true as some high-risk areas of TB were not high-risk areas for HIV). Yes, our congruence is purely visual so we changed the term from congruence to similarity.

# Reviewer 2

# Comment 1:

The revised manuscript is much improved and has sufficiently addressed my comments. Line 226 - typo in prior distribution for the intercept N(0,0). Please correct value for variance

# Revision

[Line 253] we have defined an improper flat priors for the intercept

# Comment 2:

Line 249 - additional details needed in Table 1 caption. Please define interpretation of each column e.g. 2.5th = 2.5th percentile of posterior distribution.

# Revision

[Line 282] we have amended as proposed by the reviewer

# Comment 3:

Figure 5 - can 95% credible intervals be added to this figure? This would provide the reader with further information regarding uncertainty about temporal trends.

# Revision:

Figure 5: we have adopted the suggestion from the reviewer

# Comment 4:

The scales for Figures 6 and 7(different posterior probabilities) are not the same. Changing these to a consistent scale would improve overall presentation.

# Revision:

We have removed figure seven because it is confusion.
